# Clinical manifestation of non-ketotic hyperglycemia chorea: A case report and literature review

**DOI:** 10.1097/MD.0000000000019801

**Published:** 2020-05-29

**Authors:** Weijing Wang, Xiaomei Tang, Hao Feng, Fenghui Sun, Lei Liu, Gary B. Rajah, Fengchun Yu

**Affiliations:** a Department of Neurology, Beijing Haidian Hospital, Beijing Haidian Section of Peking University Third Hospital, Beijing, P.R. China; b Department of Neurosurgery, Wayne State University School of Medicine, Detroit, MI, USA.

**Keywords:** chorea, diabetes mellitus, non-ketotic hyperglycemia

## Abstract

**Introduction:**

Chorea is considered a special complication of diabetes mellitus. Here we report a case of chorea associated with non-ketotic hyperglycemia (NKH).

**Patient concerns:**

The patient was a 79-year-old Asian woman. She had a history of type 2 diabetes mellitus more than 30 years, but with a poor control of blood sugar. She complained of acute onset of right limb involuntary activities, and being admitted to neurology department.

**Diagnosis:**

The patient was then diagnosed with NKH chorea.

**Interventions:**

Intravenous infusion of insulin was given to reduce blood glucose. Haloperidol was used to control motor symptoms.

**Outcomes:**

Her symptoms improved quickly after treatment. In the past year, the patient's blood sugar was well controlled and her chorea did not recur.

**Lessons:**

If there are sudden abnormal movements in patients, in addition to thinking of chorea, hepatolenticular degeneration and other diseases, we should also pay attention to blood sugar, especially in diabetic patients with poor blood sugar control and negative ketone, we should consider the possibility of NKK chorea.

**Conclusions:**

NKH chorea is a special complication of diabetes.

## 1 Introduction

Neurological complications of diabetes include stroke, peripheral neuropathy, epileptic seizures, etc.^[1]^ Chorea is considered a special complication and is very rare; the overall clinical features of the disease remain unknown. It is commonly seen in elderly women.^[2,3]^ Hyperglycemia,^[4–8]^ ischemia,^[9,10]^ and micro-hemorrhage^[3,11]^ may be possible mechanisms for non-ketotic hyperglycemia (NKH) chorea. In this article, we report a case of chorea associated with NKH.

## 2 Case report

A 79-year-old female complained with right limb involuntary activities for 1 day. The activity was aggravated during the day and decreased to disappear during sleep. The patient was transferred to the department of neurology for diagnosis and treatment. She had a history of type 2 diabetes mellitus for more than 18 years with poor blood glucose control. Her mother and brother also had a history of diabetes. Her father died of cerebral infarction.

**Physical examination:** her vital signs were as follows: temperature 36.5°C, blood pressure 165/95 mm Hg, respiratory rate 18/min, pulse 85 beats/min, and oxygen saturation 99% on ambient air. Neurological examination: she was somnolent with a fluent speech. Pupils were equal and reactive to light bilaterally, extra ocular movements were normal, face was symmetrical, and tongue was in midline. Left muscle tension and muscle strength were normal, right upper and lower limb muscle tension decreased, accompanied by dance-like movements, left coordination movement was stable, other physical examination found no abnormalities.

**Figure 1:**
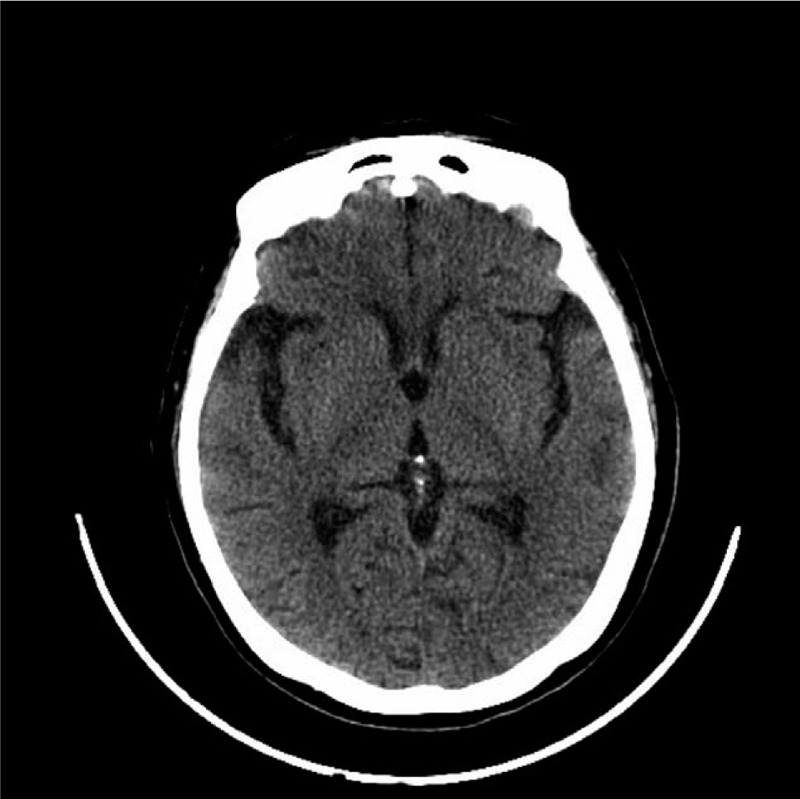
Computed-tomography of her head was negative.

**Figure 2:**
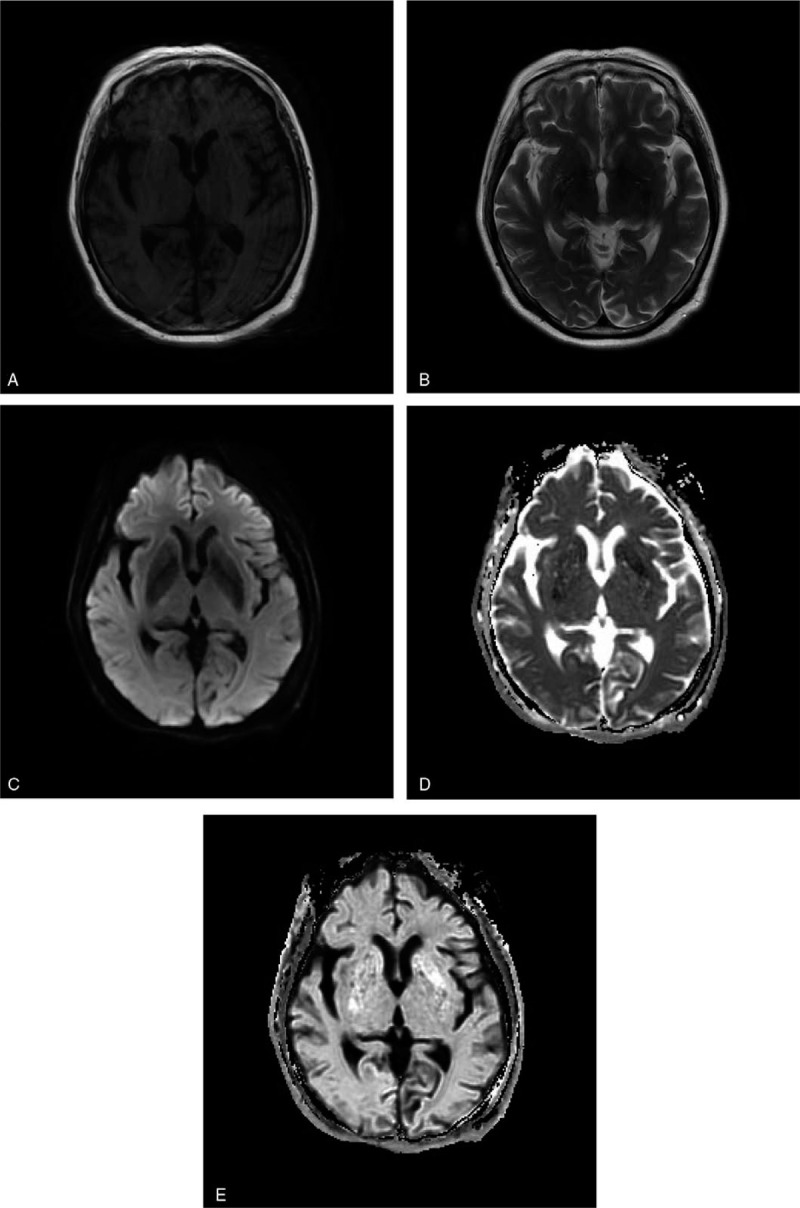
Brain magnetic resonance image (MRI) on the second day of the symptom: T1-MRI(2A), T2-MRI(2B), and DWI(2C) were normal, ADC(2D) showed low signals in the left basal ganglia, and high signals in E-ADC(2E) in the same part.

**Laboratory tests** showed that random blood glucose was 33.3 mmoL/L, glycosylated hemoglobin was 17.85%, uric sugar 4+, urinary ketone (-). Further studies including electroencephalogram, cerebrospinal fluid analysis, serum, and urine copper, ceruloplasmin, iron, ferritin, and antinuclear antibodies were unremarkable. Ophthalmologic consultation did not find KF ring. Computed-tomography (Fig. 1) of her head was negative for a cerebral infarction, cerebral hemorrhage, or brain tumor in the basal ganglia. We gave her a brain magnetic resonance image (MRI) on the second day of the symptom, T1-MRI (Fig. 2A), T2-MRI (Fig. 2B) and diffusion weighted imaging (DWI) (Fig. 2C) were normal, ADC (Fig. 2D) showed low signals in the left basal ganglia, and high signals in E-ADC (Fig. 2E) in the same part. The ADC value of left basal ganglia was 413∗10^−6^ mm^2^/s, while those of the contralateral side were normal (857∗10^−6^ mm^2^/s).

**Figure 3:**
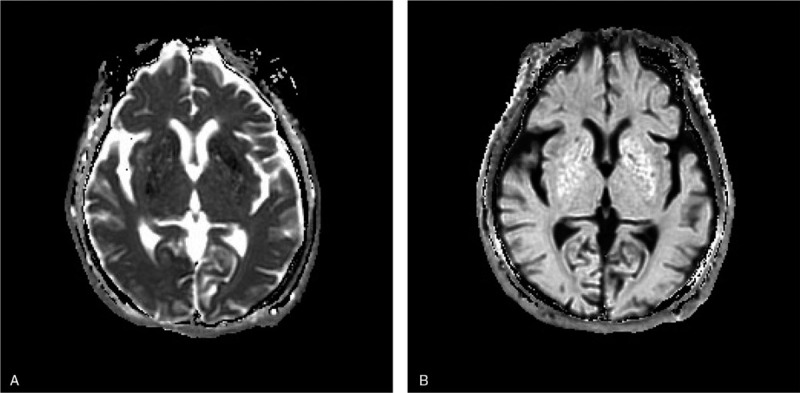
Brain magnetic resonance image after treatment: the low signals of ADC(3A) and E-ADC(3B) signal was improved than before.

**Diagnosis and treatment:** after admission, the patient was diagnosed with non-ketosis hyperglycemic chorea; intravenous infusion of insulin was given to reduce blood glucose, and intramuscular injection of 5 mg of haloperidol (HPD) per day for 3 days. After 3 days, the dance-like movement gradually decreased, and the movement range was reduced compared to that before. After the oral treatment with HPD of 2 mg twice a day, the dance-like movement of right limb gradually disappeared after 4 days. After a week, the head MRI was reviewed again: the low signals of ADC (Fig. 3A) and E-ADC (Fig. 3B) signal was improved than before. The ADC value of left basal ganglia was 625∗10^−6^ mm^2^/s, while that of the contralateral side was 842∗10^−6^ mm^2^/s.

## 3 Literature review

Neurological complications of diabetes include stroke, peripheral neuropathy, epileptic seizures, etc.^[1]^ Chorea associated with NKH chorea is considered as a special complication and is very rare. Chorea is unilateral, involuntary, irregular, wide-amplitude movement disorder, secondary to lesions in the contralateral basal ganglia. The most common etiology is an acute cerebrovascular condition, but infections, drugs, tumors, and neurodegenerative disorders can be encountered. NKH has been described as a metabolic cause of NKH chorea, especially in elderly patients with poorly controlled diabetes mellitus.^[2]^

The pathophysiology of the disease remains unclear. There are several theories about the pathological mechanism of NKH chorea. Hyperglycemia as a metabolic mechanism for NKH chorea is possible^[4–8]^; however, most metabolic disorders are bilateral (B). NKH chorea has a unilateral onset. Micro-hemorrhages may be another possible mechanism given that high signal intensity of MRI-T1 has been reported in many cases.^[12]^ The DWI and apparent diffusion coefficient (ADC) map of 1 patient displayed low ADC values in surrounding areas as well as T1 hyper intensity lesions. The ADC values were as low as in acute arterial ischemia, suggesting ischemia may be a possible mechanism of NKH chorea.^[13]^ Shan et al^[14]^ hypothesized that the possible cause of the MR imaging abnormalities might be the mild ischemia with gametocyte accumulation.

Although the clinical course of most patients is benign, not all diabetic patients will have this complication. There is currently little understanding about its pathogenesis. If we understand the pathogenesis, it is possible to avoid the occurrence of diseases. Therefore, it is beneficial to clarify the pathogenesis of this disease. To better understand the pathophysiology of this special disease entity, we performed a systematic review including all studies that involved patients with NKH who underwent a magnetic resonance imaging with DWI. The present systematic review was conducted in accordance with the Preferred Reporting Items for Systematic Reviews and Meta-Analyses Statement and Cochrane Handbook for meta-analyses and systematic reviews.

The literature search was performed by 2 authors using the several commonly used databases (from their inception to October 2018), including PubMed/Medline, Embase, the Cochrane Library, Web of Science, and China National Knowledge Infrastructure. The search strategies were the following: (“Hyperglycemia” OR “Hyperglycemia” OR “Diabetes” OR “Diabetes Mellitus”) AND (“Chorea” OR “Hemi-chorea”) AND (“MRI” OR “Magnetic Resonance Imaging”). Results were filtered to include only articles written in English or Chinese.

Two independent researchers screened and included the auto-retrieved studies in the systematic review according to the criteria described below. Eligibility studies were case reports or case series that involved patients with NKH chorea in whom a MRI with DWI scan had been performed to characterize the pathology. All studies that met the inclusion criteria were included, and studies that met the exclusion criteria were excluded.

Inclusion criteria: Researchers included studies that met the following criteria:

(1) the patients enrolled in the study were NKH chorea patients;
(2) the articles had to describe the demographic, clinical, laboratory and MRI results of the patients;
(3) the MRI result including a DWI.

Exclusion criteria: Researchers excluded studies based on the following criteria:

(1) the type of publication was not a case or case series but a review, letter, editorial, meeting abstract or others;
(2) the full text could not be obtained;
(3) humans were not subjects of the research;
(4) the article was not written in English or Chinese.

The 2 authors searched the 4 databases respectively. Database results were compared to remove duplicate results. Title and abstract screening of the articles were utilized to remove articles that were not related to the purpose of the discussion. Included articles were completely read. The 2 reviewers then compared their results to reach a consensus. A uniform collection format is designed to assess the epidemiology, clinical characteristics, laboratory, and MRI results. We found 442 articles according to the search process. Finally, only 14 articles met the requirements. Figure 1 shows the relative Preferred Reporting Items for Systematic Reviews and Meta-Analyses flow diagram. From the 14 articles, 13 patients with NKH chorea with MRI with DWI data were analyzed.^[13–23]^ (Table 1) All patients were treated in the Department of Neurology. All patients were given oral hypoglycemic drugs or intravenous insulin. Other drugs are used to control chorea, such as HPD,^[13,15,17,19,23]^ diazepam.^[15]^ None of the patients we observed used deep brain stimulation. Symptoms disappeared in most patients^[13,15–23]^; however, 1 case has left chorea and was controlled by a small dose of tiapride.^[17]^

**Table 1:**
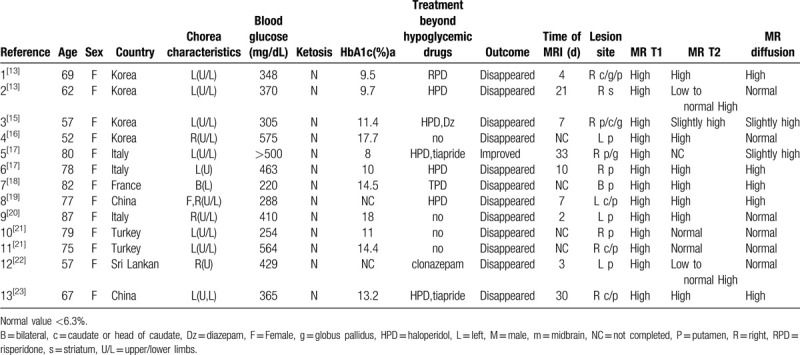
Clinical feature of the patients with non-ketotic hyperglycemia chorea.

The incidence of NKH chorea is <1/10 million, which is commonly seen in elderly women.^[12]^ Eighty percent NKH chorea patients are distributed in Asia.^[3]^ O^[12]^ 555et al conducted a meta-study in 2002. A total of 53 cases were included in the analysis. There were 30 (56.6%) women and 17 (32.1%) men (sex was not reported in 6 cases). The incidence of female is higher than that of male.

In this article, the average age was 70.9 years (52–87 years). All 13 patients (100%) were female. 9 patients (69.23%) were Asians. This suggests that older Asian women are more likely to develop NKH chorea. The conclusions are consistent with previous studies. Poor blood sugar control may lead to the disease.

## 4 Discussion

### 4.1 Pathogenesis

There are several theories about the possible mechanism of NKH chorea.

#### 4.1.1 Non-ketotic hyperglycemia as a possible mechanism

As previously reported, NKH occurs frequently in the elderly with poor blood sugar control, so hyperglycemia may be a possible mechanism for NKH chorea, in which brain metabolism changes from tricarboxylic acid circulation to anaerobic metabolism,^[4]^ which converts gamma-amino butyric acid (GABA) to succinic acid in order to provide energy. GABA can be re-synthesized in ketogenic hyperglycemia, whereas GABA and acetate are rapidly depleted in NKH.^[4]^

Therefore, acetylcholine level decreased with the depletion of acetate. Reduced GABA and acetylcholine levels in the basal ganglia may lead to functional disorders in the basal ganglia and cause the chorea symptoms.

However, with this assumption, it is difficult to explain the continuous chorea after correction of blood sugar. In some cases, Chorea also occurs in hypoglycemic^[5]^ and ketosis hyperglycemic^[5]^ patients. Some patients develop chorea^[7,8]^ after rapid correction of hyperglycemia. Most patients with NKH chorea have unilateral onset, but metabolic disorder is usually B. Therefore, these findings suggest that non ketosis hyperglycemia itself may not be the only mechanism for developing chorea.

#### 4.1.2 Ischemia as a possible mechanism

Ischemia may be a possible mechanism of NKH chorea. Kim^[9]^ reported 4 cases of hemi chorea with acute cerebral infarction. Suzuki^[10]^ reported a 66-year-old female with diabetes mellitus who suddenly developed right hemi chorea.

Her head magnetic resonance imaging (MRI) found a new cerebral infarction in the left frontal lobe, ECD-SPECT was performed on admission, and it was observed that blood flow was decreased in the left frontal lobe and striatum (s), but increased in the thalamus; 2 weeks later on a follow-up ECD-SPECT, blood flow had increased slightly in the left forebrain and s, while it had decreased slightly in the thalamus. This suggested that ischemia may be a possible of NKH chorea. However, this theory is difficult to explain the presence of B chorea in some patients.

#### 4.1.3 Micro-hemorrhage as a possible mechanism

High signal intensity of MRI-T1 has been reported in many cases,^[3]^ suggesting it may be caused by bleeding. But pathological and autopsy studies of NKH chorea^[11]^ have found s-selective neuronal loss, glial hyperplasia, and reactive astrocytosis, but no significant hematoma has been reported. Therefore, the pathogenesis of this disease needs further study.

### 4.2 Clinical manifestation

Oh^[3]^ mentioned in his research that of 53 patients with NKH chorea, 6 were B chorea (11.4%) and the other 47 were hemichorea (88.6%). Fourteen (26.4%) patients had facial involving. Most patients’ symptoms got better during sleep. In addition to chorea, some patients had other neurological deficits, such as pyramidal sign, transient dyskinesia, hemifacial spasm, and muscular dystonia.

### 4.3 Laboratory examination

Chang^[24]^ and others reported that the blood glucose levels of the 4 NKH chorea patients were much higher than the normal level. Oh^[3]^ analyzed the average blood glucose levels of the 53 patients. The average blood glucose level was 481.5 mg/dL (169–1264 mg/dL), and the HbA1c levels was 14.4% (9.9–19.2%). In our case, the female patient also reported poor blood glucose control, which accorded with the characteristics of NKH chorea patients.

### 4.4 Neuroradiological examination

MRI of NKH chorea patients showed the involvement of the putamen nucleus was common.^[3]^ The involvement of caudate nucleus and globus pallidus was rare in all cases, and the involvement of forelimbs of internal capsule and cerebral foot was also reported.^[3]^ Twelve patients with NKH chorea^[4]^ underwent routine sequence, enhanced scan, and SWI scan of the brain. All of them showed high signals in basal ganglia on T1W images, 9 cases showed slightly low signal of DWI and high signal of ADC, 3 cases showed equal signal of DWI, and no obvious change of ADC. SWI scan showed that there were stripe and low signal in the basal ganglia. After 2 weeks of treatment, abnormal signal disappeared in 7 cases, markedly reduced in 2 cases, unchanged in 1 case, and increased in 2 cases.

Normal ADC values of the parenchyma and white matter range from 780∗10^−6^ to 910∗10^−6^ mm^2^/s.^[25]^ But in our case, the ADC value of left basal ganglia was 413∗10^−6^ mm^2^/s, while that of the contralateral side was 857∗10^−6^ mm^2^/s at onset. After treatment, the ADC value of left basal ganglia was 625∗10^−6^ mm^2^/s, while that of the contralateral side was 842∗10^−6^ mm^2^/s.

The restricted diffusion of water (low ADC values) has been reported in various diseases, such as acute stroke,^[26]^ Wernicke encephalopathy,^[25]^ epidermoid mass,^[27]^ brain abscess,^[28]^ and status epilepticus.^[29]^ The proposed mechanisms of ADC decrease, which are still being discussed, including restricted diffusion, water exchange across permeable boundaries, the concept of extracellular tortuosity, and the intracellular, and extracellular volume fraction.^[30]^ In addition, while the initial triggering factors leading to restricted diffusion may vary according to the main conditions, the subsequent results may be similar, leading to cell death.

### 4.5 Treatment and prognosis

The most common treatment is blood sugar control treatment. On the basis of sugar control, HPD is used as a single therapy or combined with other drugs to control symptoms. Most of the patients given sugar control and HPD treatment of dance symptoms will be significantly improved or even disappeared.

A few serious patients, in addition to hypoglycemic treatment, can also use a variety of drugs (tipride, chlorpromazine, diazepam). For patients with intractable chorea,^[14]^ they can be treated by ventral lateral thalamotomy. Some patients relapsed after discontinuation of HPD, but relapsed patients responded well to piperazine and valproic acid.^[7]^

NKH chorea is a special complication of diabetes mellitus characterized by elevated blood glucose without ketgenesis. The incidence of NKH chorea is <1/10 million, which is commonly seen among elderly women.^[2,3]^ There are several theories about the possible mechanism of NKH chorea, such as NKH,^[4–8]^ ischemia^[9,10]^ and micro-hemorrhage.^[3,11]^ Most patients had B chorea or hemi chorea,^[18]^ some patients had facial involving.^[19]^ The patient also reported poor blood glucose control.^[13–23]^ MRI of NKH chorea patients showed the involvement of the putamen nucleus was common.^[13–23]^ The most common treatment is blood sugar control treatment.^[13–23]^ On the basis of sugar control, HPD is used as a single therapy or combined with other drugs to control symptoms.^[13,15,17,19,23]^ So far, the pathogenesis of this disease is not clear, more experimental and autopsy data and further research in larger patient series are needed to verify this hypothesis.

## Acknowledgments

We are very grateful to the patients who provided informed consent for publication of the case.

## Author contributions

**Conceptualization:** Xiaomei Tang.

**Data curation:** weijing wang, Hao Feng, Fenghui Sun.

**Formal analysis:** weijing wang, Lei Liu.

**Investigation:** Weijing Wang, Hao Feng, Fenghui Sun.

**Methodology:** Weijing Wang, Xiaomei Tang.

**Writing – original draft:** Weijing Wang, Gary B. Rajah

**Writing – review & editing:** weijing wang, Fengchun Yu.
